# Gut and sublingual microvascular effect of esmolol during septic shock in a porcine model

**DOI:** 10.1186/s13054-015-0960-3

**Published:** 2015-06-04

**Authors:** Matthias Jacquet-Lagrèze, Bernard Allaouchiche, Damien Restagno, Christian Paquet, Jean-Yves Ayoub, Jêrome Etienne, François Vandenesch, Olivier Dauwalder, Jeanne-Marie Bonnet, Stéphane Junot

**Affiliations:** Service d’anesthésie-réanimation, Hospices Civils de Lyon, Hôpital Edouard-Herriot, 5 place d’Arsonval, 69437 Lyon, Cedex 03 France; Université Claude-Bernard, Lyon 1. Campus Lyon Santé Est, 8 avenue Rockefeller, 69008 Lyon, France; EA 4174 Sepsis Inflammation Hémostase, Université de Lyon, VetAgro Sup - Campus Vétérinaire de Lyon, 1 Avenue Bourgelat, 69280 Marcy-l’Étoile, France; Laboratory of Microbiology, Groupement Hospitalier Est, 59 Boulevard Pinel, 69500 Bron, France

## Abstract

**Introduction:**

Esmolol may efficiently reduce heart rate (HR) and decrease mortality during septic shock. An improvement of microcirculation dissociated from its macrocirculatory effect may a role. The present study investigated the effect of esmolol on gut and sublingual microcirculation in a resuscitated piglet model of septic shock.

**Methods:**

Fourteen piglets, anesthetized and mechanically ventilated, received a suspension of live *Pseudomonas aeruginosa*. They were randomly assigned to two groups: the esmolol (E) group received an infusion of esmolol, started at 7.5 μg⋅kg^−1^⋅min^−1^, and progressively increased to achieve a HR below 90 beats⋅min^−1^. The control (C) group received an infusion of Ringer’s lactate solution. HR, mean arterial pressure (MAP), cardiac index (CI), stroke index (SI), systemic vascular resistance (SVR), arterio-venous blood gas and lactate were recorded. Oxygen consumption (VO_2_), delivery (DO_2_) and peripheral extraction (O_2_ER) were computed. Following an ileostomy, a laser Doppler probe was applied on ileal mucosa to monitor gut microcirculatory laser Doppler flow (GMLDF). Videomicroscopy was also used on ileal mucosa and sublingual areas to evaluate mean flow index (MFI), heterogeneity, ratio of perfused villi and proportion of perfused vessels. Resuscitation maneuvers were performed following a defined algorithm.

**Results:**

Bacterial infusion induced a significant alteration of the gut microcirculation with an increase in HR. Esmolol produced a significant time/group effect with a decrease in HR (*P* <0.004) and an increase in SVR (*P* <0.004). Time/group effect was not significant for CI and MAP, but there was a clear trend toward a decrease in CI and MAP in the E group. Time/group effect was not significant for SI, O_2_ER, DO_2_, VO_2_, GMLDF and lactate. A significant time/group effect of ileal microcirculation was found with a lower ileal villi perfusion (*P* <0.025) in the C group, and a trend toward a better MFI in the E group. No difference between both groups was found regarding microcirculatory parameters in the sublingual area.

**Conclusions:**

Esmolol provided a maintenance of microcirculation during sepsis despite its negative effects on macrocirculation. Some parameters even showed a trend toward an improvement of the microcirculation in the gut area in the esmolol group.

## Introduction

Esmolol is an ultrashort-acting beta-blocker that has been reported as an efficient treatment to decrease heart rate (HR) during septic shock; its use has been associated with reduced mortality in a recent study [1]. Despite its seemingly counterintuitive concept, this trial has confirmed a long preclinical and clinical suspicion of potential interest for the use of beta-blockers during sepsis in various animal models [2, 3], and in non-randomized study published more than four decades ago [4]. These results need further confirmation, as beta-blockade can be deleterious, especially in a context of circulatory failure where sympathetic blockade may precipitate a fatal outcome. In perioperative care, promising results [5] were not translated into survival benefit in a larger trial [6]. A better understanding of the hemodynamic effects of esmolol could permit a better timing for the initiation and to monitor treatment or to assess the eligibility of patients for this therapeutic strategy. Esmolol has been described to reduce cardiac index (CI) score, which raises the question of its immediate effect on microcirculation, particularly for the splanchnic area where early perfusion alterations occur in case of macrocirculatory failure. Besides, esmolol has a vasodilatory effect, resulting in coronary microcirculatory recruitment [7, 8]. The mechanism of this vascular effect is not univocal, but the reduction of endothelial shear stress [9] and subsequent activation of endothelial NO synthase [10] may play a role.

Esmolol has been described to enhance microcirculatory failure during sepsis [11]. However, in the study aforementioned, esmolol was administered many hours after the onset of sepsis, with no control group. Moreover, microcirculation was evaluated in the sublingual area but not in the gut area, as this area can hardly be assessed in a clinical setting. As gut and sublingual microcirculations can be uncoupled [11], and since gut barrier preservation has been described as a potential mechanism of action for beta-blockers [12], there is a need to evaluate both gut and sublingual microcirculatory during esmolol treatment in patients in critical condition. The aim of our study was to evaluate the effects of a short-acting beta-blocker at the early stages of sepsis on gut and sublingual microcirculation in a piglet model of septic shock. The primary endpoint was the effect of esmolol on gut microcirculation, evaluated by videomicroscopy. The secondary endpoint was the effect of esmolol on sublingual microcirculation, macrocirculation, oxygen metabolism and lactate.

## Materials and methods

### Animal preparation

The study was approved by our local ethical board for animal research and care (Vet Agro Sup, Marcy l’Etoile, France, authorization number: 1252) in accordance with European regulations (Directive EU 86/609). A total of 14 healthy female piglets, weighing between 25 and 40 kg and two to three-months-old, were selected. At 12 h before the beginning of the experimental phase solid food was withdrawn, while access to water was still allowed. After sedation with an intramuscular administration of tiletamine/zolazepam (Zoletil 100, 100 mg⋅mL^−1^, Virbac, Carros, France), 3.0 mg⋅kg^−1^ and morphine 0.2 mg⋅kg^−1^, induction was carried out with an intravenous administration of propofol (PropoFlo 10 mg/mL, Axience, Pantin, France) 4.0 mg⋅kg^−1^, and maintained with isoflurane (Vetflurane, Virbac Carros France) given to effect in 30 % oxygen, morphine (0.1 mg⋅kg^−1^⋅h^−1^) and cisatracurium (0.15 mg ⋅kg^−1^⋅h^−1^).

Animals were orotracheally intubated and mechanically ventilated. A pulse oximeter was placed on the tongue to continuously measure hemoglobin saturation (SpO_2_) and HR. An electrocardiogram continuously monitored the heart by means of three electrodes placed on the thorax of the animals. Several catheters were surgically inserted after inguinal incision and lateral cervicotomy. An arterial catheter was inserted into the right femoral artery for systemic arterial pressure (MAP) measurement. A central venous catheter was inserted in the femoral vein for fluid and drug administration and central venous pressure (CVP) measurement. A pulmonary arterial catheter (Edwards Life science® catheter, Irvine, USA) was inserted in the right jugular vein for mean pulmonary arterial pressure (MPAP), pulmonary capillary wedge pressure and cardiac (CO) output measurements. The suitable location of the catheter tip was determined by the characteristic of the pressure curve. An ileostomy was performed to allow positioning of a laser Doppler probe (Perimed Instrument, Järfälla-Stockholm, Sweden) fixed on a balloon to enable a contact between the probe and the mucosa. Multiple videomicroscopy samples were carried out on the stoma site. The abdomen and the neck wounds were then sutured (PDS II, Ethicon, Issy-les-Moulineaux, France). Blood samples were analyzed with a blood gas analyzer (ABL90 Flex, Radiometer, Neuilly-Plaisance, France).

### Experimental protocol

**Fig. 1:**
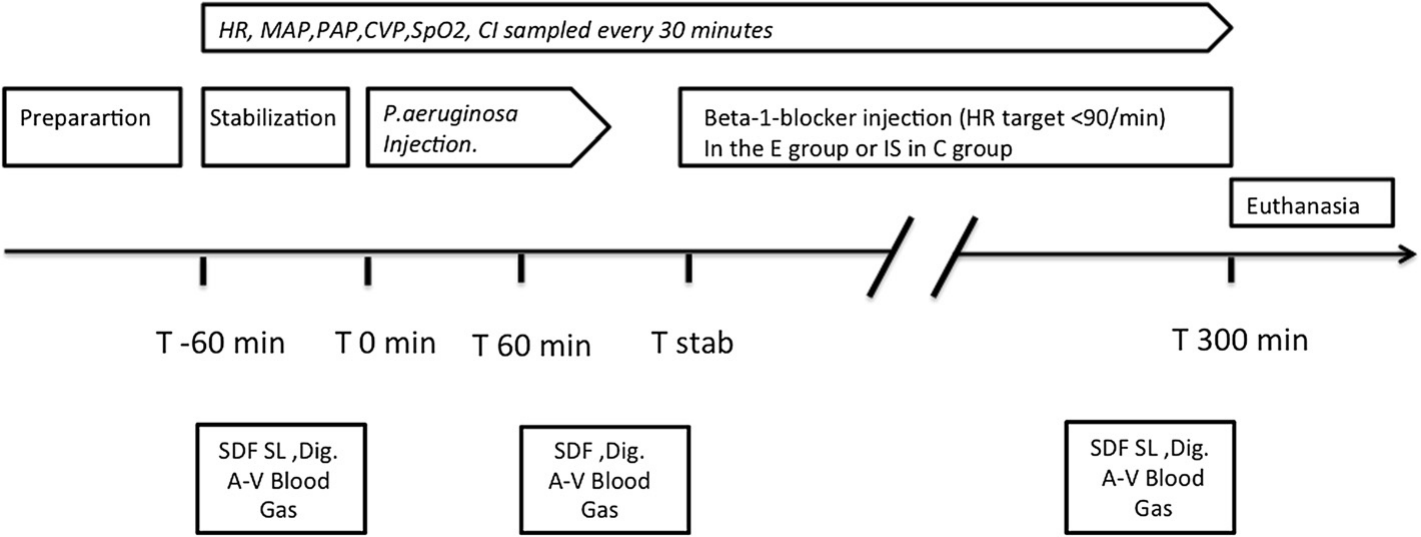
Study design. After the stabilization period, animals received an intravenous infusion at T 0 min of *Pseudomonas aeruginosa*, 0.3 mL⋅20 kg^−1^⋅min^−1^. At the onset of shock, resuscitation was started until stabilization (T stab), around 90 min after T 0 min. Animals were then randomized to receive esmolol in the E group; esmolol was started (or isotonic saline (IS) in the C group) at 7.5 μg⋅kg^−1^⋅min^−1^ and increased until HR target (<90 min^−1^) was reached. The experiment was ended after 300 min. A-V Blood Gas, arterio-venous blood gas; CI, cardiac index; CVP, central venous pressure; HR, heart rate; MAP, systemic mean arterial blood pressure; MPAP, mean pulmonary arterial blood pressure; SpO_2_, pulsed oxygen saturation

The experimental procedure is detailed in Fig. 1. After a baseline period of 60 min, every piglet received an infusion of *Pseudomonas aeruginosa* bacteria (Laboratoire de microbiologie du groupe hospitalier est, Hospice civil de lyon. Bron , France) (5 × 10^8^ colony forming unit (CFU)⋅mL^−1^ perfused at 0.3 mL⋅20 kg^−1^⋅min^−1^). The systolic pulmonary arterial pressure (SPAP) was monitored. When SPAP reached 45 mmHg, the bacterial infusion was stopped to limit the right ventricular afterload and avoid any right heart dysfunction. The goal was to obtain a reproducible hyperdynamic septic shock as originally described [13, 14]. After initiation of resuscitation and hemodynamic stabilization, animals were randomly assigned to two groups: esmolol group (E) and control group (C). Animals of the control group (n = 6) received an infusion of Ringer’s lactate solution alone; those of the E group (n = 6) also received an infusion of esmolol.

Esmolol was started at 7.5 μg⋅kg^−1^⋅min^−1^ (Baxter, Maurepas, France), and progressively increased to decrease HR to between 80 and 90 beats⋅min^−1^. Intravenous Ringer’s lactate (Aguettant, Lyon, France) was administered during the experiment at a basal rate of 10 mL⋅kg^−1^⋅h^−1^. Resuscitation procedures were performed as follows: objectives were to maintain MAP above 60 mmHg, venous saturation in oxygen (SvO_2_) above 70 % and CI above 80 % of its basal value. A fluid load of 250 mL of Ringer’s lactate solution was administered initially to evaluate fluid responsiveness; in case of absence of fluid response, norepinephrine (if the MAP objective were not fulfilled) and/or milrinone (if the CI objective was not achieved and if there was no fluid responsiveness) were used. The initial dose of norepinephrine (Renaudin, Itxassou, France) was 0.04 μg⋅kg^−1^⋅min^−1^. The initial dose of milrinone (Sanofi, Paris, France) was 0.375 μg⋅kg^−1^⋅min^−1^, and could be increased to up to 0.75 μg⋅kg^−1^⋅min^−1^ to achieve resuscitation goals. At the end of the experimental time, animals were killed using embutramide 0,3 ml/kg of a mixture of embutramide, mebezonium and tetracaine T61 (200mg ofembutramide, 26.92 mg of mebezonium and 4.39 mg of tetracaïne per ml), Intervet, Beaucouze, France.

### Bacterial preparation

A *P. aeruginosa* reference strain (ATCC 27853) was used to induce septic shock. This strain was kept in a glycerolized heart/brain broth at −80 °C at the microbiology laboratory (Laboratoire de Bactériologie du Centre de Biologie et Pathologie Est, Hospices Civils de Lyon, France). At 48 h before the experiment, two subcultures were systematically performed to avoid any variation of the reference strain. The suspension was kept in a refrigerator, ready for use less than two hours before the administration into the piglets. To check the inoculum concentration, two dilutions of bacteria suspension were streaked on MH agar plates by Spiral instrument (AES/bioMérieux, Marcy l’Etoile, France). These plates were read after overnight incubation at 37 °C and compared to nomogram of the Spiral® system to obtain the concentration of bacteria suspension.

### Data collection

HR, MAP, MPAP, CVP and, SpO_2_ were continuously monitored. Cardiac output was measured every 30 min and assessed three times by thermodilution technique with 10 mL of cold solute injected *via* the pulmonary arterial catheter. Arterial and venous blood gases were analyzed every 60 min. Standard formula were used to compute CI (L⋅min^−1^/⋅m^−2^), stroke index ((SI) mL⋅min^−1^⋅m^−2^), systemic vascular resistance (SVR), pulmonary vascular resistance ((PVR) dyn⋅s⋅cm^−5^⋅m^−2^), oxygen delivery ((DO_2_) L⋅min^−1^⋅m^−2^) and consumption ((VO2) L⋅min^−1^⋅m^−2^). This enabled us to evaluate DO2/VO2 dependency by calculating the correlation coefficient and oxygen extraction ratio ((O_2_ER) %). Body surface area was calculated as previously described [15].

The laser Doppler probe provided a signal of gut perfusion named gut microcirculatory laser Doppler flow (GMLDF). Videomicroscopy measurements of the sublingual and gut microcirculation were obtained at T-60 min, T 60 min (before bacterial injection, after bacterial injection and before esmolol administration) and T 270 min (after esmolol administration) using a sidestream dark-field (SDF) imaging device (Microscan, Microvision medical, Amsterdam, The Netherlands) with a × 5 lens. Images of the microcirculation were obtained for the ileal mucosa through the ileostomy, and for the sublingual area. The camera was applied without pressure, after removal of secretions. Five sequences of five to 20 s each, from different areas were recorded. A blinded investigator (MJL) performed the semi-quantitative analysis of the sequences. For the sublingual area, only vessels that crossed one of the nine lines used to assess the De Backer score were considered and scored as perfused or not, enabling calculation of the proportion of total perfused vessels ((PPV total) %), small vessels perfused ((PPV small vessels) %) and other vessels perfused ((PPV other vessels) %). Small vessels were defined as a diameter smaller than 20 μm. Other vessels were defined as a diameter larger than 20 μm. Microvascular flow was qualitatively evaluated (from 0 to 3) for each of the four quadrants and each site; microvascular mean flow index (MFI) was calculated by using an ordinal scale with 0 meaning no flow, 1 an intermittent flow, 2 a sluggish flow and 3 a normal flow [16, 17].

Heterogeneity index was calculated as previously described [18]: an absolute change of 10 % in the PPV small vessels was considered as clinically significant [19]. For intestinal area, the SDF camera’s objective was inserted through the ileostomy to the depth of 5 to 7 cm from the edge of the stoma, with a slight angulation as previously described [20]. Heterogeneity index was calculated as the difference between the highest and lowest MFI, divided by the mean MFI of all sites at a single time point. PV was estimated as the percentage of perfused villi divided by the total number of villi or crypts times 100 % [16].

### Statistical analysis

Statistical analysis was performed with R software (R-project, GNU GPL.) [21]. We used several packages of the CRAN R project and computed descriptive statistics for all data [22]. We used a Kolmogorov-Smirnov test to check the normality of variables distribution. If the distribution was not normal, data were expressed as median and interquartile range (25^th^ to 75th percentiles) for every variable, as appropriate. We used an analysis of variance (ANOVA) test with repeated measures to detect if there was any effect of time or group on the different variables. Our data were not normally distributed, but we checked that they were close to a normal distribution on the frequency histogram, as ANOVA is not very sensitive to moderate deviations from normality [23–25]. The Mann–Whitney *U* test and Wilcoxon test were performed as appropriate. If data were too different at baseline, data were expressed as variations from baseline and expressed in percentage of variation from baseline. Spearman’s correlations were used to evaluate the relationship between hemodynamic and microcirculation variables. Statistical significance was defined as a *P* value lower than 0.05.

## Results

A total of 14 piglets were initially selected, but two piglets died before randomization and were withdrawn from the study. The analysis was finally composed of 12 pigs: six in the E group and six in the C group, weighing a mean of 37.3 kg (range: 35.6 to 39.2), with a mean of 0.79 m^2^ (range: 0.77 to 0.82 m^2^) body surface area. Weight of the piglets appeared relatively similar between groups: the mean weight in the E group was 36.5 kg (range: 36.0 to 37.3 kg) and the mean weight in the C group was 38.2 kg (range: 34.6.6 to 40.0 kg); however, we noticed a significant difference between both groups (Wilcoxon test, *P* = 0.047). Similarly, mean body surface areas were 0.78 m^2^ (range: 0.77 to 0.79 m^2^) in the E group and 0.80 m^2^ (0.75 to 0.83 m^2^) in the C group (*P* = 0.047). The maximal limit of SPAP (45 mmHg threshold to stop bacterial infusion) was reached within a mean of 27.0 min (range: 23.5 to 41.0 min) in the C group and 37.0 min (range: 34.0 to 40.0 min) in the E group. These durations of bacterial administration were not significantly different between both groups (*P* = 0.410).

### Effect of bacterial infusion on microcirculation and macrocirculation in both groups

Hemodynamic effects of *P. aeruginosa* infusion on micro and macrocirculation are shown in Table 1. Data were compared between T-60 and T 60 time points for all piglets. Resuscitation maneuvers had already started for every piglet at T 60, but esmolol administration had not started at that time. A significant increase in HR and a trend towards a significant increase in MAP and CI were observed.

**Table 1:** Effect of bacterial infusion on hemodynamic and metabolic parameters

	–T-60 min	T 60 min	*P* value
Hemodynamic			
HR (min^−1^)	97 (90 - 103)	117 (104 - 136)	0.007
MAP (mmHg)	64.9 (57.9 - 71.4)	71.2 (65.8 - 74.5)	0.106
MPAP (mmHg)	16.2 (14.9 - 19.1)	35.9 (31.5 - 38.8)	0.002
PCWP (mmHg)	7.6 (5.5 - 10.5)	8.6 (6.6 - 11.0)	0.307
CVP (mmHg)	9.9 (8.3 - 11.4)	11.3 (7.9 - 12.8)	1
CI (L⋅min^−1^⋅m^−2^)	2.4 (2.2 - 2.7)	2.9 (1.9 - 3.02)	0.62
SI (mL⋅m^−2^)	24.6 (23.4 - 27.3)	21.1 (18.0 - 27.8)	0.151
SVR (dyn⋅s⋅cm^5^⋅m^−2^)	2,458 (1,943 - 2,604)	2,344 (1,787 - 2,869)	0.577
PVR (dyn⋅s⋅cm^5^⋅m^−2^)	161.6 (149.3 - 191)	358.7 (314.9 - 388.8)	0.002
Metabolic			
Lactate (mmol⋅L^−1^)	2.1 (1.8 - 2.8)	1.7 (1.4 - 2.8)	0.045
O_2_ER (%)	14.0 (8.9 - 19.1)	14.1 (11.3 - 15.8)	0.432
O_2_ER/CI	5.5 (3.5 - 7.4)	4.8 (2.9 - 6.4)	0.622
PaO_2_/FiO_2_ (mmHg)	327.8 (253.3 - 436.4)	385.2 (240.4 - 422.3)	0.470
Gut microcirculation			
*Videomicroscopy*			
MFI of the gut	2.7 (2.5 - 2.9)	1.56 (1.25 - 1.75)	0.003
Heterogeneity index of the gut (%)	17.7 (4.7 - 33.4)	61.9 (27.5 - 72.3)	<0.001
Ratio of perfused villi (%)	94.2 (90.0 - 97.7)	68.1 (59.0 - 76.5)	0.005
*laser Doppler flow*			
GMLDF (ua)	154.2 (85.9 - 699.6)	130.7 (80.15 - 674.7)	1
GMLDF variation	1.04 (0.87 - 1.08)	0.92 (0.85 - 1.18)	0.677
Sublingual microcirculation			
TVD (n⋅mm)	17.7 (15.8 - 18.4)	11.7 (11.2 - 12.9)	0.021
MFI	2.8 (2.7 - 2.9)	2.2 (2.1 - 2.3)	<0.001
MFI small vessels	2.7 (2.5 - 2.9)	1.6 (1.4 - 1.8)	0.003
MFI other vessels	3.0 (2.9 - 3.0)	2.8 (2.8 - 2.9)	0.036
Heterogeneity index (%)	13.2 (4.4 - 24.2)	64.6 (34.4 - 84.2)	<0.001
PPV other vessels	99.7 (96.2 - 100)	96.03 (75.6 - 100)	0.262
PPV small vessels	91.9 (88.2 - 95.2)	64.6 (51.9 - 68.1)	<0.001

Hemodynamic parameters before and after the onset of sepsis but before esmolol administration. Wilcoxon test were performed to compare variables before and after sepsis. *HR:* heart rate, *MAP*: systemic mean arterial blood pressure, *MPAP*: mean pulmonary mean arterial blood pressure, *PCWP*: pulmonary capillary wedge pressure, *CVP*: Central venous pressure, *SI*: stroke index computed as CI/HR, *SVR:* systemic vascular resistance, *CI*: cardiac index, *PVR*: pulmonary vascular resistance, *O*_*2*_ *ER*: oxygen extraction ratio, *FiO*_*2*_ *:* fraction of inspired oxygen, *GMLDF*: gut microcirculatory laser Doppler flow, *TVD*: total vessels density, *MFI*: microvascular flow index. Ratio of perfused villi: proportion of perfused villi, PPV: proportion of perfused vessels, small vessels: vessels with a diameter under 20 μm, Other vessels: vessels with a diameter above 20 μm, mmHg: millimeters of mercury, mmol⋅L^−1^: millimoles⋅Liter, PaO_2_: arterial partial pressure of oxygen

In parallel, a significant alteration of microcirculation in the gut area was observed with a significant decrease in MFI and PV, and a significant increase in heterogeneity. An alteration of sublingual microcirculation was also found with a significant decrease in total vessels density (TVD), MFI, heterogeneity index and PPV small vessels (Table 1). No significant difference between groups was found at that time.

### Effect of esmolol on macrocirculation

**Fig. 2:**
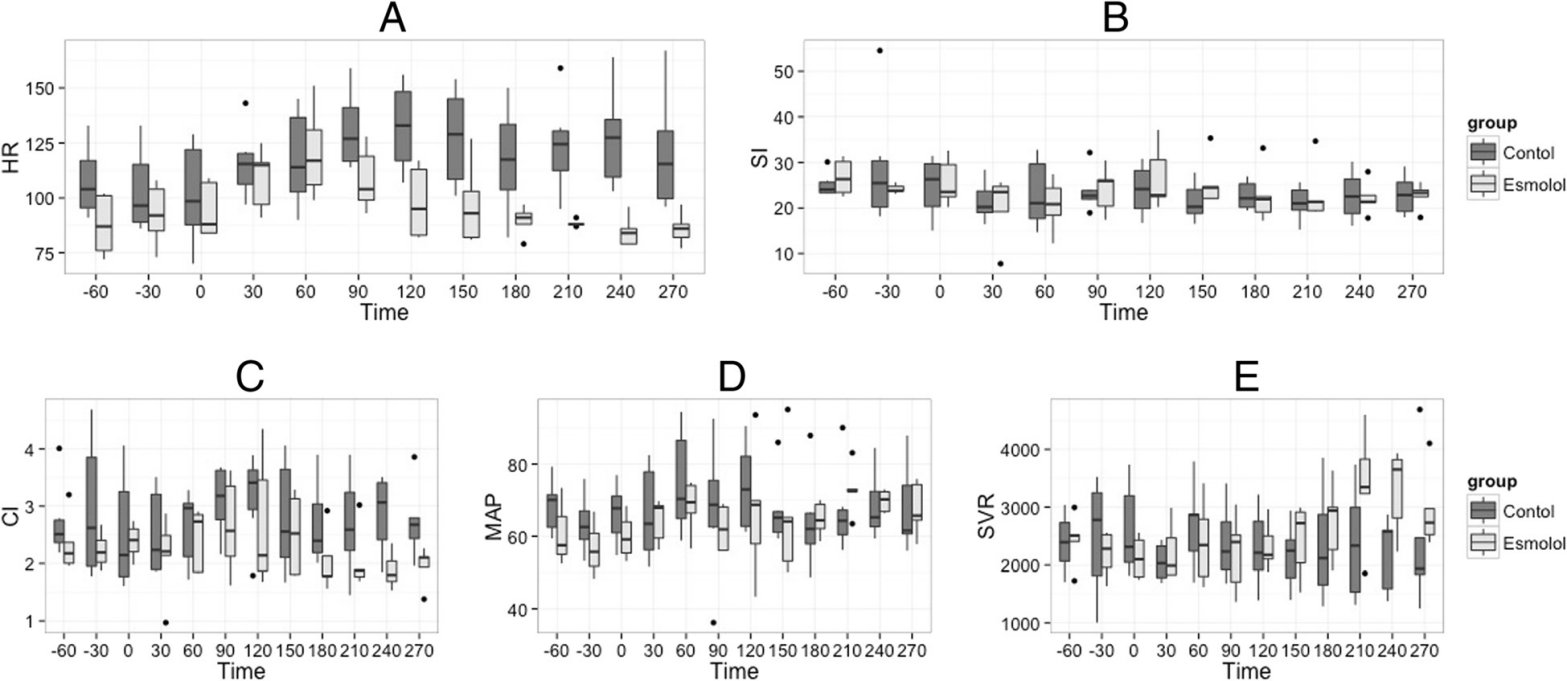
Evolution of hemodynamic variables during the experiment. T-60 min is the beginning of stabilization; T 0 min is the time of bacterial injection. Esmolol is started and hemodynamic stabilization occurs between T 60 and T 180 min after resuscitation is started, and the experiment concluded at T 300 min. ANOVA were performed for each parameter: (**a**) HR, group effect *P* <0.001, time effect *P* = 0.223, time/group effect *P* = 0.004; (**b**) SI, group effect *P* = 0.222, time effect *P* = 0.050, time/group effect *P* = 0.404; (**c**) CI, group effect *P* = 0.006, time effect *P* = 0.508, time/group effect *P* = 0.339; (**d**) MAP, group effect *P* = 0.099, time effect *P* = 0.042, time/group effect *P* = 0.051; (**e)** SVR, group effect *P* = 0.662, time effect *P* = 0.023, time/group effect *P* = 0.004; HR, heart rate; CI, cardiac index; SI, stroke index computed as CI/HR; MAP, systemic mean arterial blood pressure; SI, stroke index computed as CI/HR; SVR, systemic vascular resistance

Hemodynamic stabilization was obtained between T 60 min and T 180 min (prerequisite for esmolol injection). Results are shown in Fig. 2. We observed a significant time/group effect with a decrease in HR as well as a trend toward a decrease in MAP and in CI. We observed a significant increase in SVR in the esmolol group. SI was not significantly different between both groups. We did not observe any significant time/group effect for the evolution of PVR (Fig. 2).

### Metabolic effect of esmolol

**Fig. 3:**
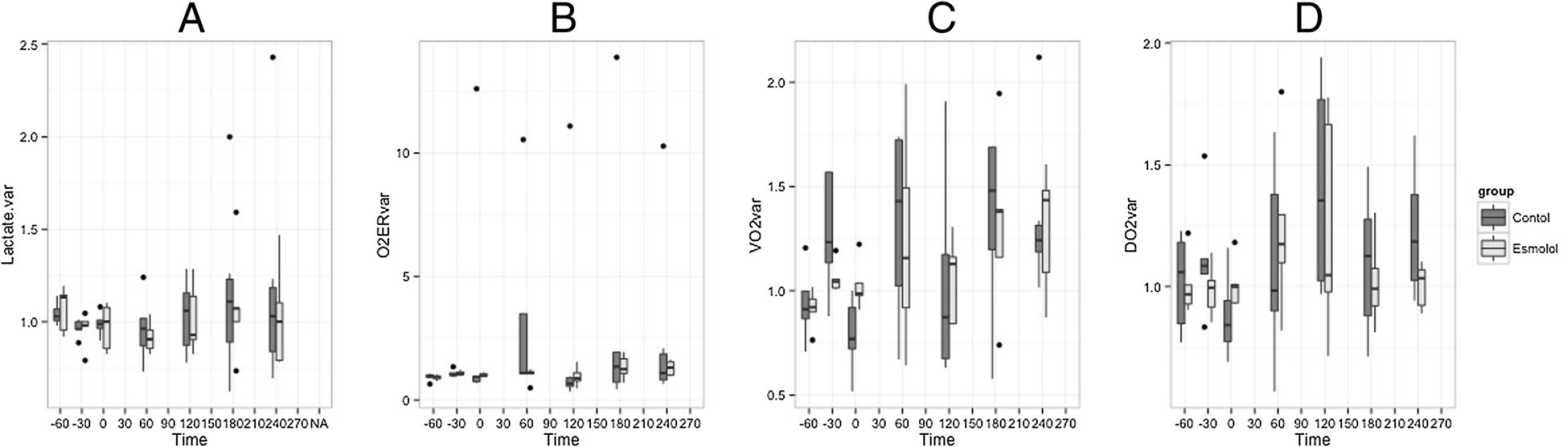
Evolution of metabolic variables during the experiment. T-60 min is the beginning of stabilization; T 0 min is the time of bacterial injection. Esmolol is started and hemodynamic stabilization occurs between T 120 and T 180 min, and the experiment concluded at T 300 min. ANOVA were performed for each parameter: (**a**) Lactate, group effect *P* = 0.486, time effect *P* = 0.117, time/group effect *P* = 0.398; (**b**) O_2_ERvar, group effect *P* = 0.026, time effect *P* = 0.203, time/group effect *P* = 0.423; (**c**) VO_2_var, group effect *P* = 0.710, time effect *P* <0.001, time/group effect *P* = 0.510; (**d**) DO_2_var, group effect *P* = 0.823, time effect *P* = 0.062, time/group effect *P* = 0.470. DO_2_, VO_2_, O_2_ER and lactate are expressed as variation (variation are computed as t_x_ divided by t_baseline_). Lactate.var, lactate variation, O_2_ERvar, oxygen extraction ratio computed as the difference between arterial oxygen saturation and central venous oxygen saturation divided by arterial oxygen saturation, VO_2_var, oxygen consumption, DO_2_var, oxygen delivery

Results of the metabolic effect of esmolol are shown in Fig. 3. DO_2_ and VO_2_ did not vary significantly in the E group. There was a manifest trend toward a significant difference between E and C group for the DO_2_. No significant difference between groups was observed for lactate. There was no DO_2_/VO_2_ dependency, as attested by the poor correlation between DO_2_ and VO_2_ in both groups (rDO_2_/VO_2_ = 0.367; *P* = 0.001). This correlation was not significant in the E group (DO_2_/VO_2_ = 0.263; *P* = 0.093). Median value of SvO_2_ remained between 77 and 85 % in each group throughout the experiment. No significant time effect (*P* = 0.446) or time/group effect (*P* = 0.119) was found, while a significant group effect was found (*P* = 0.029).

### Effect of esmolol on microcirculation

**Fig. 4:**
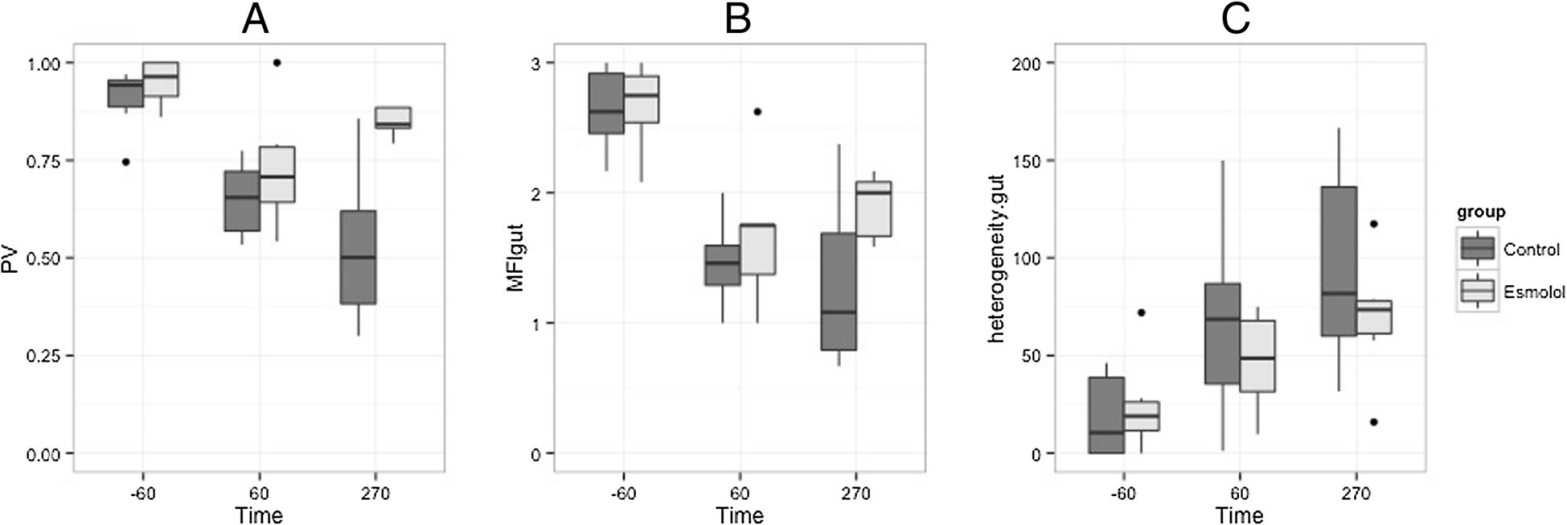
Evolution of gut microcirculation variables during the experiment. T-60 min is the beginning of stabilization; T 0 min is the time of bacterial injection. Esmolol is started and hemodynamic stabilization occurs between T 60 and T 180 min after resuscitation is started, and the experiment concluded at T 300 min. ANOVA were performed for each parameter: results are detailed in Table 2. (**a)** A significant time/group effect was found for the ratio of perfused villi. No significant effect was found for (**b**) MFI gut or (**c**) heterogeneity.gut. PV, perfused villi ratio is defined as the ratio of perfused villi under the number of villi visible on the stabilized video; MFI gut, Mean Flow Index of the ileal mucosa; heterogeneity.gut, heterogeneity of the ileal mucosa computed as the difference between the highest and lowest MFI, divided by the mean MFI of all sites at a single time point; GMLDF, Gut microcirculatory laser Doppler blood flow

The effects of esmolol on gut microcirculation are detailed in Fig. 4. Regarding gut area, a significant difference in evolution of PV was found between both groups (time/group effect, *P* = 0.025) with a non significant increase in MFI for the E group (time/group effect, *P* = 0.2) (Table 2 and Fig. 4). Concerning sublingual area, MFI was not significantly different between groups. No significant effect of esmolol on sublingual microcirculation was observed (time/group effect, *P* = 0.08). GMLDF did not significantly differ between both groups. We observed a dissociation between the gut and sublingual microcirculation as suggested by the moderate correlation between the parameters of the two localizations (rMFI small vessels-MFI gut = 0.608, *P* = 0,001; r heterogeneity sublingual-heterogeneity gut = 0.126, *P* = 0.538). We did not find a significant correlation between videomicroscopy parameters and GMLDF.

**Table 2:** Effect of esmolol on microcirculation of the E group compared to the C group

Time	−60 min				60 min				270 min				Group effect	Time effect	Time/group effect
Group	Control		Esmolol		Control		Esmolol		Control		Esmolol		*P* value	*P* value	*P* value
*Gut*															
MFI of the gut	2.6	(2.5 - 2.9)	2.8	(2.5 - 2.9)	1.5	(1.3 - 1.6)	1.8	(1.4 - 1.8)	1.1	(0.8 - 1.7)	2	(1.7 - 2.1)	0.129	<0.001	0.200
Ratio of perfused villi	0.9	(0.8 - 1)	1	(0.9 - 1)	0.7	(0.6 - 0.7)	0.7	(0.6 - 0.8)	0.5	(0.4 - 0.7)	0.8	(0.8 - 0.9)	0.006	<0.001	0.025
Heterogeneity index of the gut	10.3	(3 - 8.7)	18.8	(11.4 - 26.1)	68.6	(35.4 - 86.9)	48.6	(31.4 - 67.9)	81.8	(60 - 136.4)	73.5	(61.3 - 78)	0.267	0.001	0.376
*Sublingual*															
TVD (n⋅mm)	18	(17.6 - 18.3)	16.1	(14.9 - 17.8)	12,9	(11,7 - 14,2)	11.4	(11.1 - 11.8)	15.9	(14.7 - 17)	15.9	(15.6 - 15.6)	0.270	0.822	0.931
TVD small vessels (n⋅mm)	14.7	(14.2 - 15.6)	12.3	(12.3 - 12.6)	11.8	(11.1 - 13.7)	10.3	(9.8 - 11.5)	12.8	(12.1 - 14.3)	12.2	(12.1 - 13.7)	0.074	0.262	0.141
TVD other vessels (n⋅mm)	2.4	(2 - 3)	3.4	(2.4 - 4.4)	0,6	(0,5 - 1,3)	1.0	(0.8 - 1.2)	2.5	(2 - 2.9)	3	(2.2 - 3.4)	0.022	0.074	0.151
PPV small vessels	94.1	(92.7 - 95.5)	87.3	(75.2 - 89.8)	63.7	(55.2 - 68.1)	64.6	(49.8 - 67.6)	75.5	(71.9 - 80.4)	77.5	(69.1 - 88.4)	0.568	0.183	0.212
PPV other vessels	100	(98.6 - 100)	98	(95.5 - 99.9)	92.7	(68.4 - 98.7)	98.5	(84.5 - 99.9)	97.4	(88.6 - 100)	96.8	(84.4 - 100)	0.772	0.63	0.757
MFI small vessels	2.8	(2.6 - 2.9)	2.7	(2.5 - 2.8)	1.7	(1.6 - 2.0)	1.5	(1.3 - 1.7)	2	(1.9 - 2.3)	2.1	(1.6 - 2.6)	0.528	0.07	0.542
MFI other vessels	3	(3 - 3)	2.9	(2.8 - 3)	2.8	(2.8 - 2.9)	2.9	(2.8 - 2.9)	2.8	(2.7 - 2.9)	2.8	(2.7 - 3)	0.552	0.051	0.491
Heterogeneity index	8.9	(4.4 - 17.1)	18	(6.5 - 26.8)	56.8	(38.6 - 97.4)	72.8	(37.8 - 78.9)	42	(29 - 60.7)	26.1	(18.2 - 33.5)	0.401	0.012	0.08

Gut microcirculatory parameters were performed on the ileal mucosa through a stoma. Sublingual microcirculation was measured at the base of the tongue. The cut-off value to differentiate small vessels and other vessels is 20 μm. Density of vessel is performed by the De Backer score: six lines cross the image and three vertical and three horizontal vessels that cross the line are considered. The total of vessels crossing lines over total line length shows the density of vessels

TVD, Total vessels density; MFI of the gut, Mean Flow Index of the ileal mucosa; MFI small vessels, sublingual Mean Flow Index of small vessels; MFI other vessels, Mean Flow Index of other vessels; Density small vessels, density of small vessels; Density other vessels, density of other vessels; PPV small vessels, proportion of perfused vessels for small vessels; PPV other vessels, proportion of perfused vessels for other vessels

### Effect of esmolol on resuscitation requirements

The effect of esmolol on resuscitation requirements are shown in Table 3. A trend toward a greater amount of resuscitation was observed with more fluid in the E group, but these results were not significant (*P* = 0.093). A significantly greater amount of milrinone was administered in the E group, but only one piglet required this drug to fulfill the resuscitation criteria.

**Table 3:** Drug and fluid administration in the esmolol and control group

	E group	C group	*P* value
Fluids (mL)	625 (500 - 938)	250 (250 - 438)	0.093
Norepinephrine (μg)	2130 (1469 - 2127)	1833 (1167 - 2250)	0.484
Esmolol (mg)	180 (160 - 1840)	0 (0 - 0)	0.003
Milrinone (mg)	0 (0 - 1.81)	0 (0 - 0)	0.016

E group*,* esmolol group; C group, control group

## Discussion

In our experimental model of septic shock, esmolol administration induced a significant decrease in HR and a trend towards a decrease in CI and MAP. In contrast to these macrocirculatory effects, esmolol did not alter sublingual and gut microcirculation; conversely, a small improvement of some of the microcirculatory parameters was observed.

The use of beta-blockers during septic shock has been associated with a decreased mortality [1], but the reasons for this remain to be elucidated. Among their potential interesting effects, beta-blockers have been associated with a modulation of immunity, hemodynamic and metabolic disorders induced by sepsis [26]. Esmolol has also been reported to prevent gut barrier dysfunction [12, 27], an effect that seems attractive as the gut is suspected to play a major role in the sustainability of sepsis, with its dysfunction leading to multiple organ dysfunction syndrome [28]. The immunologic effect is unclear, but different studies have reported a diminution of systemic inflammation and a diminution of cytokines decompartmentalization [3]. Beta-blockers may also re-orientate cytokines from an adrenergic-induced pro-inflammatory Th2-type profile to an anti-inflammatory Th1-type profile [26, 29]. Esmolol may also provide cardioprotective effects by improving myocardial DO_2_ and cardiac output [2]. Finally, its short half-life permits a better compliance of the treatment in a context of circulatory failure [30]. Recently, this drug has been reported to improve septic shock mortality in humans [1].

In our experiment, microcirculatory impairments were observed at the onset of shock. As an early resuscitation was administered, we could not observe any lactate elevation, prolonged hypotension or low cardiac output. Even though the increase in CI and MAP were not significant, the trends of these macrocirculatory parameters (with a significant increase in HR) were in favor of hyperdynamic shock. These cardiovascular modifications were associated with a decrease in microcirculatory indexes. Such an uncoupling of macro and microcirculation during sepsis is consistent with the literature [31, 32], and is associated with worsened outcome [17].

Esmolol was given after stabilization of macrocirculatory parameters. As expected, it produced a significant decrease in HR, as well as a trend towards a significant decrease of MAP and CI, and an increase in SVR. This rise of SVR can be explained by the greater amount of norepinephrine used in the E group, even though this difference was not significant; it could also be the result of a mathematical coupling between a decrease of CI and a maintenance of MAP. Another explanation could be the preservation of the vasoreactivity by esmolol. The effects of esmolol on SVR are not consistent in the literature: it has been reported to increase [1] or decrease SVR [33]. We did not observe any improvement in SI or CI in the present study, and milrinone was used in only one piglet in the E group to fulfill the therapeutic target. This result was unexpected as an increase in SI was anticipated, related to a better ventricular filling. In a non-resuscitated rat model, an increase in SI was described and attributed to an improvement of preload and a longer diastole [29].

Such an improvement was also found by Morelli *et al.* in human patients, but an inotropic drug (levosimendan) was administered in both treated and control groups [1]. The increased use of milrinone in our study was consistent with a previous study using metoprolol [34] and another study using atenolol, where no effect on SVR, CI and oxygen consumption were reported [35]. We assume that when enough fluid is administered for hemodynamic resuscitation of sepsis, there is no preload dependency for cardiac output, as predicted by the Frank-Starling law of the heart. Thus, even if preload is increased by a longer diastole, it does not necessarily increase cardiac output. This could explain why our results are different from other models of septic shock described in previous studies where no fluid were administered [29].

No reduction of VO_2_ was observed in the E group, which differs from the results of Morelli *et al.* [1], but is consistent with a previous study performed on septic piglets where the effect of esmolol was assessed earlier during the time course of sepsis [33]. Considering its potential microcirculatory recruitment, esmolol should increase VO_2_; however, this effect is dampened by a decreased HR, cardiac oxygen consumption and hyper-adrenergic cell catabolism, which may reduce VO_2_.

In our experiment, despite macrocirculation impairment, we did not observe a significant alteration of microcirculation following esmolol administration. Conversely to the control group, gut microcirculation parameters, assessed with SDF, remained stable. Some of the gut microcirculatory parameters improved in the E group.

The mechanisms of these positive effects on microcirculation remain to be determined. The beta theory of shock developed by Berk *et al.* [4] has never gained acceptance. In this theory, treatment with beta-blockers may induce a reduction of shunting and subsequent microcirculatory recruitments. An increase in SI may also restore pulsatility and reverse microcirculation impairment in near laminar flow [36], as previously reported in some studies [1, 33]. This is not supported by our experimental data, as SI was not increased in the E group. Other non-hemodynamic effects of beta-blockers can be considered: beta-1-selective adrenergic receptor blockers have been reported to increase endothelial NO production, which may facilitate microcirculation recruitment [37]. A fibrinolytic activity may also dampen the pro-coagulant state induced by sepsis [38], and prevent microcirculation impairment related to microthrombi formation [39].

Evaluation of correlation between gut and sublingual microcirculation was not the principal aim of our study, and we report no correlation between both microcirculations in our experimental setting, which remains consistent with the conflicting reports in the literature regarding the link between both sites [40, 41]. Nevertheless, microcirculatory evolution trends appeared quite similar between both sites in our data.

We acknowledge some limitations to our study. First, our model of septic shock did not meet all the criteria of hyperdynamic shock, as there was only a trend toward significance for the observed increase in CI. This could be due to a lack of statistical power due to the small sample size. Second, we noticed a difference between both groups at baseline for some variables, which may also be attributed to the small sampling size of our experimental setting. Weight and body surface area were different between groups: even though these differences were statistically significant, they were not considered clinically relevant as the difference was only 1.7 kg. This difference was also minimized by the fact that all data was indexed by body weight or by body surface area. Third, SvO_2_ appeared different between both groups. Even if there was a significant group effect, it does not seem relevant as all data remained in normal ranges. Moreover, no time/group effect was found, which is in favor of an absence of effect of esmolol on SvO_2_ evolution. The conclusion that we drew on DO_2_ and consumption is hampered by the fact that our method of calculation of these parameters may be biased due to mathematical coupling of the data. Calorimetry could have been used as an alternative method, but its utilization is cumbersome, with its own limitations [42].

Fourth, we observed a slight elevation of lactate and a slight impairment of microcirculation at the beginning of the experiment, followed by a normalization during the stabilization period. This may be explained by surgical preparation [43], induction of anaesthesia before endotracheal intubation or drugs used for anesthesia induction [44]. Fifth, the lack of correlation between laser Doppler and videomicroscopy was unexpected, but may be explained by the fact that the sampling depth of the laser Doppler signal is much deeper compared with videomicroscopy. A large part of the laser Doppler signal is thus related to the arteriolar component. Only a small component of microcirculation with vessels less than 20 μm (which are analyzed in the villi) seemed to be influenced by sepsis. Thus, any effect of esmolol may have been dampened with laser Doppler technology. To our knowledge, videomicroscopy and laser Doppler have not been formally compared. Sixth, we did not assess organ dysfunction and survival to sepsis, but the study was not designed for this purpose. We cannot exclude that the better microcirculation observed in the E group may be related to the greater amount of fluids and positive inotropes and/or norepinephrine administered, even though the differences between groups were not significant. An increased administration of fluids has been reported to enhance microcirculation at the early phase of sepsis [45]. Finally, the dose of esmolol used and the target chosen may have been too aggressive and could also partially explain the negative effect on CI, which was not observed in previous studies. However, there is not enough data in the literature to evaluate the suitable doses and targets. The introduction of esmolol occurred very early in the course of sepsis compared to other studies, and we do not know if the effects of esmolol observed in our experimental conditions are sustainable for a longer period.

## Conclusions

Esmolol provided a maintenance of microcirculation during sepsis despite its effects on macrocirculation. Some of our data suggest an improvement in gut microcirculation. A trend towards a decrease in CI and DO_2_ was observed following esmolol administration, but lactate and VO_2_ were not significantly altered. These results provide a better understanding of the hemodynamic and microcirculatory effects of beta-blockers during sepsis, with a decreased uncoupling between microcirculation and macrocirculation, even though the mechanism of action remains to be elucidated. The fear of a negative microcirculatory effect of esmolol is not supported by our data, but this treatment should be carefully developed in the time course of sepsis. In addition to macrocirculation monitoring, assessing microcirculation could be an interesting guide to evaluating the appropriate dosage and timing of administration of esmolol.

## Key messages

Esmolol allowed better maintenance of gut microcirculation despite a reduction of stroke index in a resuscitated piglet model of septic shock.

## Abbreviations

CI: Cardiac index
CVP: Central venous pressure
DO_2_: Oxygen delivery
GMLDF: Gut microcirculatory laser Doppler flow
HR: Heart rate
MAP: Mean arterial pressure
MFI: Mean flow index
MPAP: mean pulmonary arterial pressure
O_2_ER: Oxygen extraction ratio
PPV: Proportion of perfused vessels
PV: Perfused villi
PVR: Pulmonary vascular resistance
SDF: Sidestream dark-field
SI: Stroke index
SPAP: Systolic pulmonary arterial pressure
SpO_2_: hemoglobin saturation
SvO_2_: Venous saturation in oxygen
SVR: Systemic vascular resistance
TVD: Total vessels density
